# New Insights into Plant Signaling Mechanisms in Biotic and Abiotic Stress

**DOI:** 10.3390/plants14131953

**Published:** 2025-06-26

**Authors:** Hamdy Kashtoh, Muhammad Fazle Rabbee, Kwang-Hyun Baek

**Affiliations:** Department of Biotechnology, Yeungnam University, Gyeongsan 38541, Republic of Korea; hamdy_kashtoh@ynu.ac.kr (H.K.); rabbi_biotech@ynu.ac.kr (M.F.R.)

Plants are constantly challenged by their environments, including both biotic and abiotic stress factors. As a result, plants have developed complex signaling pathways in response to various challenges, allowing them to adapt and survive [1]. To detect and react to pathogen attacks, herbivore feeding, and symbiotic interactions in the case of biotic stress, plants use a complex network of signaling molecules, including phytohormones, reactive oxygen species (ROS), and secondary metabolites [2,3]. These signaling cascades cause the activation of systemic acquired resistance, the synthesis of antimicrobial chemicals, the reinforcement of physical barriers, and genes involved in defense. When plants are exposed to abiotic stress, such as extreme temperatures, drought, salinity, and nutrient deficiencies, they use different signaling pathways to adapt [4,5]. Abscisic acid, ethylene, jasmonic acid, calcium ions (Ca^2+^), and other signaling molecules are involved in these pathways [6]. These signaling molecules coordinate cellular responses such as stomatal closure, osmotic correction, and the activation of stress-responsive genes. Understanding the mechanisms of plant signaling networks involved in biotic and abiotic stress responses is essential for developing crop plants that are resilient to changing environmental conditions [7]. This Special Issue aims to present recent contributions to developing our understanding of the mechanisms involved in plant responses to biotic and abiotic stress. It features ten papers, comprising four reviews and six research studies that address the aforementioned aspects.

In this Special Issue, four articles discussed the tolerance mechanisms that plants exert to adapt to stress and highlighted their stress signaling networks. One study summarized the challenges faced by rice (*Oryza sativa* L.) due to global climate change, which induces various abiotic stresses that detrimentally affect rice grain quality and yield. The study highlighted the defensive strategies rice plants employ to deal with abiotic stressors, particularly drought, salinity, submergence, extreme temperatures, and heavy metal toxicity, which significantly influence key morphological, chemical, and metabolic processes. Furthermore, it also outlined approaches for developing rice cultivars that can endure multiple abiotic stresses [8]. Another article addressed the issue of microbial pathogens that impede the growth of plants and their productivity. It described how plants identify pathogens, effectors, and microbe-associated molecular patterns (PAMPs or MAMPs) as imminent danger signals and initiate various immune responses like effector-triggered immunity (ETI) and PAMP-triggered immunity (PTI) [9]. Additionally, it also discussed the roles of autophagy, RNA silencing, and systemic acquired immunity as dynamic host-mediated defensive responses against pathogens. Moreover, it underlined the initial biochemical signaling processes, including ROS, Ca^2+^, and hormones, that activate various plant immune response mechanisms.

Two manuscripts provided a comprehensive analysis of stress signaling molecules and their role in plant stress adaptation. One of them elaborated on the importance of the role of plant heterotrimeric G protein signaling in maintaining a balance between normal growth and stress adaptation. It illustrated the signaling pathways by which heterotrimeric G proteins assist plants in regulating growth, while also adapting to immune challenges and thermomorphogenesis [10]. The second article discussed another signaling molecule that has a pivotal role in stress signaling networks, the plant-specific protein kinase, sucrose non-fermenting-1-related protein kinase 2 (SnRK2). This kinase plays a crucial part in helping plants adapt to stress by phosphorylating downstream targets, which in turn influences gene expression and physiological responses [11]. This article explored the substrates that SnRK2 phosphorylates in *Arabidopsis thaliana*, providing a comprehensive understanding of their roles in stress signaling and developmental processes. Furthermore, it described various post-translational modifications (PTMs) that SnRK2 undergoes, which collectively fine-tune its stability, activity, and intracellular dynamics, demonstrating an intricate feedback system that manages the activation and attenuation of the kinase.

Six manuscripts covered a variety of topics, including plants’ responses to salt, alkaline, high-temperature stress, and pathogen infection, using different analytical tools such as genomics, transcriptomics, proteomics, and metabolomics approaches to understand such responses. One interesting article discussed the modulating roles of MYC2 and BBX21 transcriptional factors (TFs) in the flavonoid network in plants [12]. The study suggested that MYC2 has a dual role (activator/repressor) in regulating the anthocyanin pathway, depending on the cellular environment. Additionally, there is a possibility that BBX21 plays a similar role in the regulation of the *BAN* gene within the proanthocyanidin pathway in both *O. sativa* and *Arabidopsis thaliana*. Another article investigated the way in which wheat plants manage root exudation in response to alkali stress, employing a metabolomics method to detect and quantify root exudates generated in these circumstances. The research concentrated on transcriptional and metabolic processes, particularly alkali stress-induced secreted metabolites (AISMs) [13]. The findings suggested that when wheat plants are under alkali stress, the release of multiple metabolites containing a –COOH group plays a vital role in pH regulation. In response to alkali stress, wheat plants increase the synthesis of fatty acids, glycolysis, and phenolic acid production, which will supply additional energy and substrates for root exudation. Similarly, another study conducted on celery (*Apium graveliens* L.) investigated the synergistic effects of aspartic acid (Asp) and silicon (Si) in mitigating salt stress. The study showed that salt toxicity, which is identified via an altered nutritious status, hindered photosynthetic ability, reduced plant growth, and disrupted internal ion balance, and that an activated antioxidant defense system (indicated by higher levels of antioxidant enzymes and lower ROS accumulation) was ameliorated through the use of Si, Asp, or a combination of both [14]. Importantly, the combined application of Si and Asp was found to be more effective in minimizing salt stress compared to applying either of them individually. In summary, the exogenous application of Si and Asp aided in alleviating salt stress and enhanced the salt tolerance of celery.

Another interesting article described how a plant systemically responds to stress or stimulus by means of variation potential (VP). The study focused on the mechanisms that influence the specificity of VP in response to different local stimuli, including heating, burning, and wounding, which all result in distinct VP parameters [15]. It suggested that the varying functions of hydraulic and chemical signals determine the distinct characteristics of these VP parameters. The phenomenon by which VP triggers systemic responses is likely linked to variations in the concentration of ions, such as Ca^2+^ and H^+^, that occur during the generation of VP. Overall, these findings indicated that the specificity of VP in response to stimuli stems from the unique properties of the chemical and hydraulic signals that create it, which may additionally impact variations in ion concentrations.

To study plant–pathogen interactions and their effects on plant growth, Li et al. investigated how *GhSTR1*, a member of the ABCG subfamily of ATP-binding cassette (ABC) transporters, mediates the defense mechanisms of cotton (*Gossypium hirsutum*) plants against various pathogens [16]. The study suggested that *GhSTR1* plays a role in cotton’s defense against Verticillium wilt and Fusarium wilt, which are caused by the fungal pathogens *Verticillium dahliae* and *Fusarium oxysporum.* These fungi infect the plant’s vascular system, resulting in wilting, yellowing, and often plant death. The study also suggested that *GhSTR1* mediates the plant’s vegetative and reproductive development, seemingly balancing the trade-off between defending against pathogens and promoting plant growth.

Expression analysis is a powerful tool for deciphering the regulatory mechanisms of different genes during plants’ environmental stress responses. In this Special Issue, one study examined the *PIN* gene family, auxin efflux transporter proteins, and identified nine members of the *CsPIN* gene family in the cucumber (*Cucumis sativus* L.) genome [17]. Furthermore, it investigated the expression levels of *CsPIN* genes in both leaves and roots when subjected to different abiotic stresses and hormone treatments. Different *CsPIN* genes showed varied response patterns to abiotic stresses like NaCl, high temperature, and PEG, as well as to different hormone signals, aiding in the regulation of auxin balance and facilitating plant adaptation to environmental changes.

In summary, this Special Issue features a series of research studies that deepen our knowledge of the fundamental mechanisms underlying plant responses to different stresses. Gaining insight into these defense mechanisms contributes to the development of effective strategies aimed at boosting plant resilience and productivity in harsh conditions.

## References

[1] Suzuki N., Rivero R.M., Shulaev V., Blumwald E., Mittler R. (2014). Abiotic and biotic stress combinations. New Phytol..

[2] Rejeb I., Pastor V., Mauch-Mani B. (2014). Plant Responses to Simultaneous Biotic and Abiotic Stress: Molecular Mechanisms. Plants.

[3] Demidchik V. (2015). Mechanisms of oxidative stress in plants: From classical chemistry to cell biology. Environ. Exp. Bot..

[4] Kashtoh H., Baek K.-H. (2021). Structural and Functional Insights into the Role of Guard Cell Ion Channels in Abiotic Stress-Induced Stomatal Closure. Plants.

[5] Deng Y., Kashtoh H., Wang Q., Zhen G., Li Q., Tang L., Gao H., Zhang C., Qin L., Su M. (2021). Structure and activity of SLAC1 channels for stomatal signaling in leaves. Proc. Natl. Acad. Sci. USA.

[6] Zhang H., Zhu J., Gong Z., Zhu J.-K. (2022). Abiotic stress responses in plants. Nat. Rev. Genet..

[7] Cramer G.R., Urano K., Delrot S., Pezzotti M., Shinozaki K. (2011). Effects of abiotic stress on plants: A systems biology perspective. BMC Plant Biol..

[8] Sarma B., Kashtoh H., Lama Tamang T., Bhattacharyya P.N., Mohanta Y.K., Baek K.H. (2023). Abiotic Stress in Rice: Visiting the Physiological Response and Its Tolerance Mechanisms. Plants.

[9] Ali S., Tyagi A., Mir Z.A. (2024). Plant Immunity: At the Crossroads of Pathogen Perception and Defense Response. Plants.

[10] Lee H. (2024). Trade-Off Regulation in Plant Growth and Stress Responses Through the Role of Heterotrimeric G Protein Signaling. Plants.

[11] Wei Y., Peng L., Zhou X. (2025). SnRK2s: Kinases or Substrates?. Plants.

[12] Li N., Xu Y., Lu Y. (2024). A Regulatory Mechanism on Pathways: Modulating Roles of MYC2 and BBX21 in the Flavonoid Network. Plants.

[13] Wang H., Zhao S., Qi Z., Yang C., Ding D., Xiao B., Wang S., Yang C. (2024). Regulation of Root Exudation in Wheat Plants in Response to Alkali Stress. Plants.

[14] Song J., Yang J., Jeong B.R. (2024). Synergistic Effects of Silicon and Aspartic Acid on the Alleviation of Salt Stress in Celery (*Apium graveliens* L.) “Si Ji Xiao Xiang Qin”. Plants.

[15] Mudrilov M., Ladeynova M., Vetrova Y., Vodeneev V. (2024). Analysis of the Mechanisms Underlying the Specificity of the Variation Potential Induced by Different Stimuli. Plants.

[16] Cheng G., Li X., Fernando W.G.D., Bibi S., Liang C., Bi Y., Liu X., Li Y. (2025). Fatty Acid ABCG Transporter GhSTR1 Mediates Resistance to Verticillium dahliae and Fusarium oxysporum in Cotton. Plants.

[17] Zhang Y., Zhu K., Shen W., Cui J., Miao C., Lu P., Wu S., Zhu C., Jin H., Zhang H. (2025). The PIN Gene Family in Cucumber (*Cucumis sativus* L.): Genome-Wide Identification and Gene Expression Analysis in Phytohormone and Abiotic Stress Response. Plants.

